# The genome sequence of the Crescent Bell, *Epinotia bilunana* (Haworth, 1811)

**DOI:** 10.12688/wellcomeopenres.19334.1

**Published:** 2023-04-12

**Authors:** Douglas Boyes, James Hammond, University of Oxford and Wytham Woods Genome Acquisition Lab, Darwin Tree of Life Barcoding collective, Wellcome Sanger Institute Tree of Life programme, Wellcome Sanger Institute Scientific Operations: DNA Pipelines collective, Tree of Life Core Informatics collective, Darwin Tree of Life Consortium

**Affiliations:** 1 UK Centre for Ecology & Hydrology, Wallingford, England, UK; 2 University of Oxford, Oxford, England, UK

**Keywords:** Epinotia bilunana, Crescent Bell, genome sequence, chromosomal, Lepidoptera

## Abstract

We present a genome assembly from an individual male *Epinotia bilunana* (the Crescent Bell; Arthropoda; Insecta; Lepidoptera; Tortricidae). The genome sequence is 659.0 megabases in span. Most of the assembly is scaffolded into 28 chromosomal pseudomolecules, including the Z sex chromosome. The mitochondrial genome has also been assembled and is 15.4 kilobases in length.

## Species taxonomy

Eukaryota; Metazoa; Ecdysozoa; Arthropoda; Hexapoda; Insecta; Pterygota; Neoptera; Endopterygota; Lepidoptera; Glossata; Ditrysia; Tortricoidea; Tortricidae; Olethreutinae; Eucosmini; *Epinotia*; *Epinotia bilunana* (Haworth, 1811) (NCBI:txid1594293).

## Background

*Epinotia bilunana* (Haworth, 1811) is a moth of the Tortricidae family. This species is widely distributed across the British Isles and northern Eurasia ( Bradley *et al.*, 1979; Elliott *et al.*, 2018; GBIF Secretariat, 2022), being found almost anywhere with birch ( *Betula*) woodland.

The larvae feed from September to April within the catkin of birches, sometimes betraying their presence by distorting the catkin ( Bradley *et al.*, 1979; Elliott *et al.*, 2018). Pupation occurs in May either within the larval feeding site or in a silken cocoon amongst leaf litter ( Bradley *et al.,* 1979; Elliott *et al.*, 2018). Adults are on the wing from late May to September from dusk onwards, but are readily disturbed from birch foliage and trunks by day ( Bradley *et al.*, 1979; Elliott *et al.*, 2018). The forewings of the adult moth are typically a light grey or creamy white with black markings, but can show variation in the strength of black colouration ( Bradley *et al.*, 1979).

The genome of *Epinotia bilunana* was sequenced as part of the Darwin Tree of Life Project, a collaborative effort to sequence all named eukaryotic species in the Atlantic Archipelago of Britain and Ireland. Here we present a chromosomally complete genome sequence for *Epinotia bilunana*, based on one male specimen from Wytham Woods, Oxfordshire, UK.

## Genome sequence report

**Figure 1.**
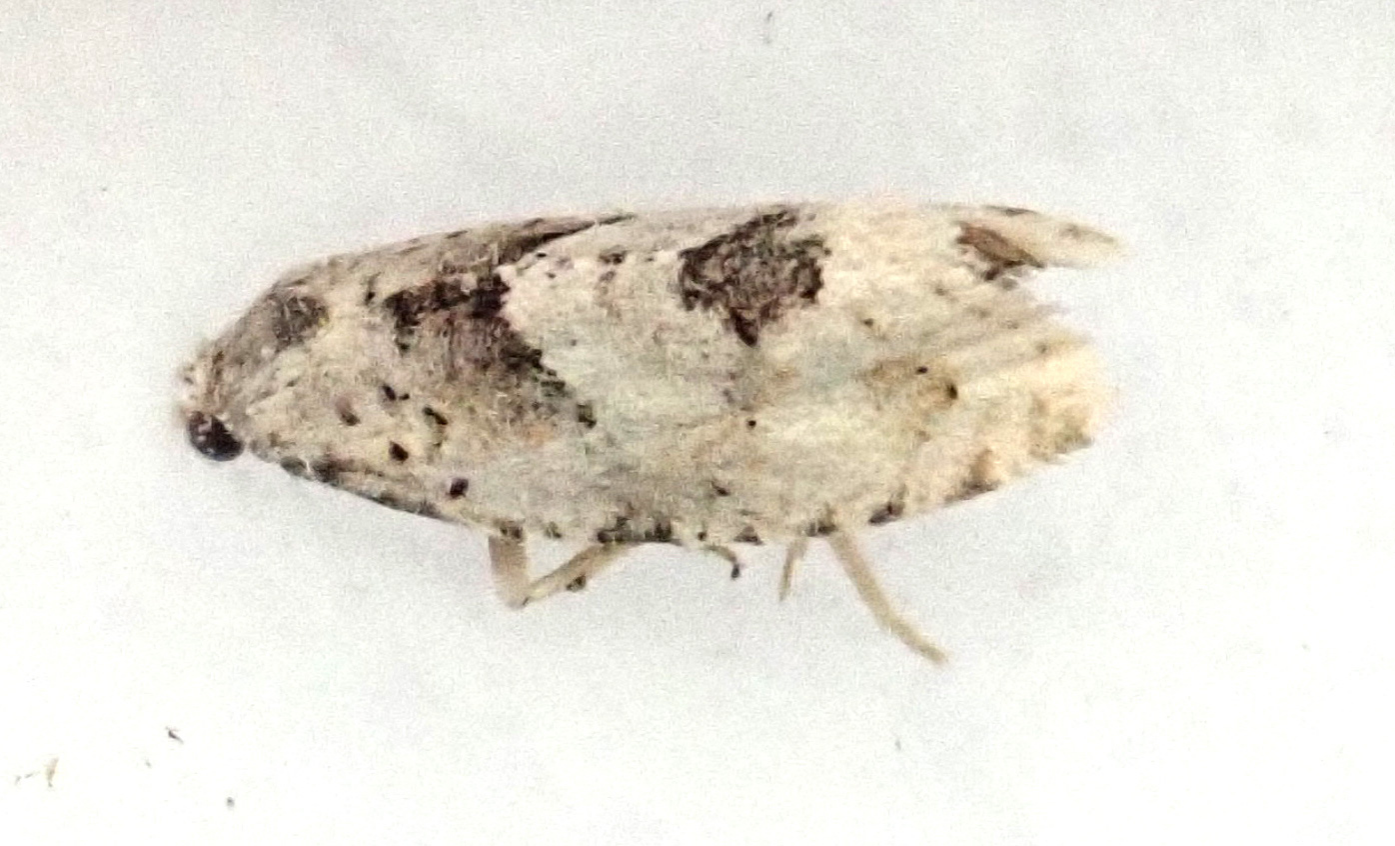
Photograph of the *Epinotia bilunana* (ilEpiBilu1) specimen used for genome sequencing.

The genome was sequenced from one male *Epinotia bilunana* ( Figure 1) collected from Wytham Woods, UK (latitude 51.77, longitude –1.34). A total of 28-fold coverage in Pacific Biosciences single-molecule HiFi long reads was generated. Primary assembly contigs were scaffolded with chromosome conformation Hi-C data. Manual assembly curation corrected 16 missing joins or mis-joins and removed five haplotypic duplications, reducing the assembly length by 0.48% and the scaffold number by 4.17%.

**Figure 2.**
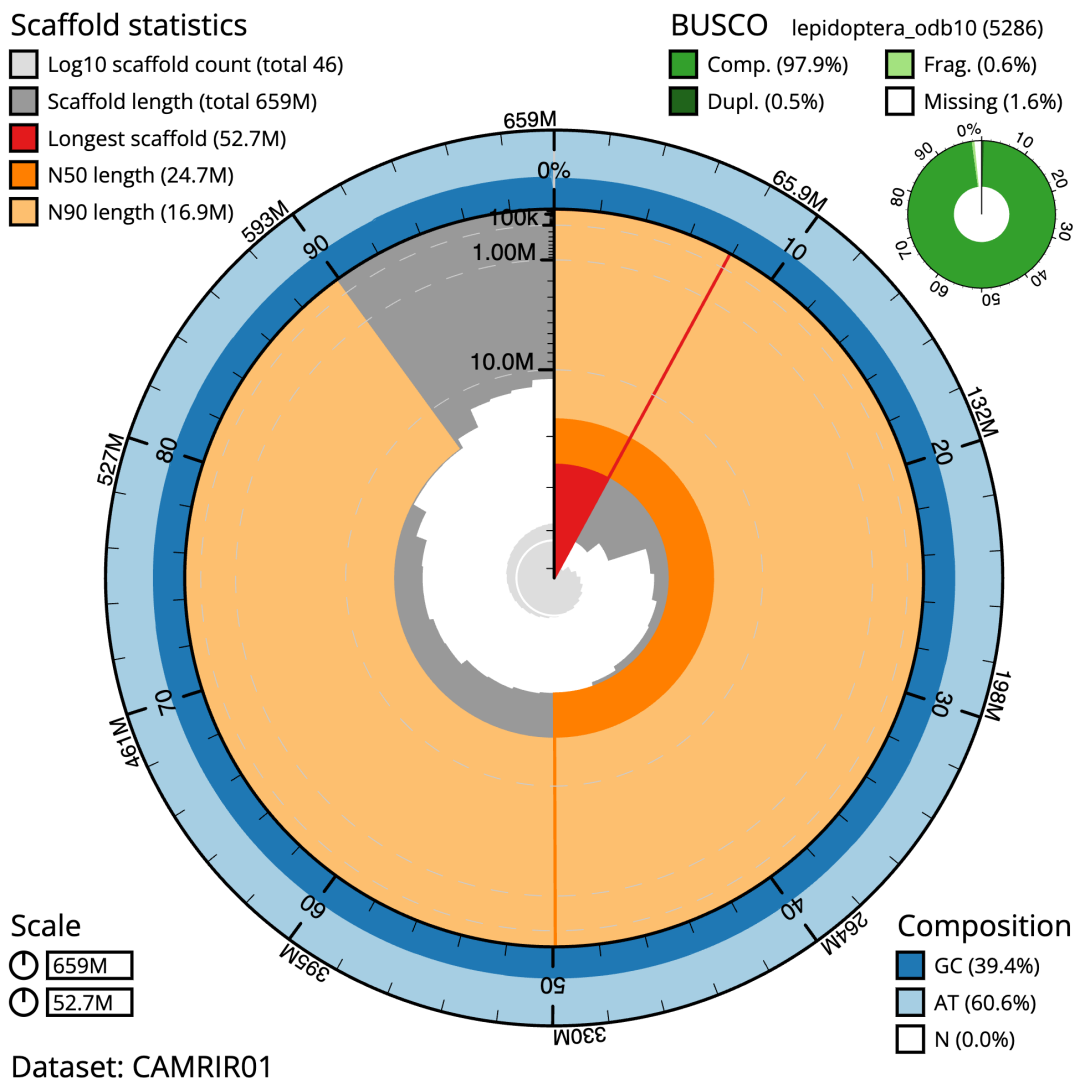
Genome assembly of *Epinotia bilunana*, ilEpiBilu1.1: metrics. The BlobToolKit Snailplot shows N50 metrics and BUSCO gene completeness. The main plot is divided into 1,000 size-ordered bins around the circumference with each bin representing 0.1% of the 659,043,568 bp assembly. The distribution of scaffold lengths is shown in dark grey with the plot radius scaled to the longest scaffold present in the assembly (52,658,368 bp, shown in red). Orange and pale-orange arcs show the N50 and N90 scaffold lengths (24,745,286 and 16,921,967 bp), respectively. The pale grey spiral shows the cumulative scaffold count on a log scale with white scale lines showing successive orders of magnitude. The blue and pale-blue area around the outside of the plot shows the distribution of GC, AT and N percentages in the same bins as the inner plot. A summary of complete, fragmented, duplicated and missing BUSCO genes in the lepidoptera_odb10 set is shown in the top right. An interactive version of this figure is available at https://blobtoolkit.genomehubs.org/view/ilEpiBilu1.1/dataset/CAMRIR01/snail.

The final assembly has a total length of 659.0 Mb in 46 sequence scaffolds with a scaffold N50 of 247.5 Mb ( Table 1). Most (99.82%) of the assembly sequence was assigned to 28 chromosomal-level scaffolds, representing 27 autosomes, and the Z sex chromosome. Chromosome-scale scaffolds confirmed by the Hi-C data are named in order of size ( Figure 2– Figure 5; Table 2). While not fully phased, the assembly deposited is of one haplotype. Contigs corresponding to the second haplotype have also been deposited. The mitochondrial genome was also assembled and can be found as a contig within the multifasta file of the genome submission.

**Table 1.** Genome data for *Epinotia bilunana*, ilEpiBilu1.1.

Project accession data		
Assembly identifier	ilEpiBilu1.1	
Species	*Epinotia bilunana*	
Specimen	ilEpiBilu1	
NCBI taxonomy ID	1594293	
BioProject	PRJEB55885	
BioSample ID	SAMEA10979172	
Isolate information	ilEpiBilu1, male: whole organism (genome sequencing); ilEpiBilu2, male: whole organism (Hi-C scaffolding)	
Assembly metrics*		*Benchmark*
Consensus quality (QV)	64.7	*≥ 50*
*k*-mer completeness	100%	*≥ 95%*
BUSCO**	C:97.9%[S:97.4%,D:0.5%], F:0.6%,M:1.6%,n:5,286	*C ≥ 95%*
Percentage of assembly mapped to chromosomes	99.82%	*≥ 95%*
Sex chromosomes	Z chromosome	*localised homologous pairs*
Organelles	Mitochondrial genome assembled	*complete single alleles*
Raw data accessions		
PacificBiosciences SEQUEL II	ERR10224852	
Hi-C Illumina	ERR10177758	
Genome assembly		
Assembly accession	GCA_947049275.1	
*Accession of alternate haplotype*	GCA_947049265.1	
Span (Mb)	659.0	
Number of contigs	143	
Contig N50 length (Mb)	8.3	
Number of scaffolds	46	
Scaffold N50 length (Mb)	24.7	
Longest scaffold (Mb)	52.7	

* Assembly metric benchmarks are adapted from column VGP-2020 of “Table 1: Proposed standards and metrics for defining genome assembly quality” from ( Rhie *et al.*, 2021). ** BUSCO scores based on the lepidoptera_odb10 BUSCO set using v5.3.2. C = complete [S = single copy, D = duplicated], F = fragmented, M = missing, n = number of orthologues in comparison. A full set of BUSCO scores is available at https://blobtoolkit.genomehubs.org/view/ilEpiBilu1.1/dataset/CAMRIR01/busco.

**Figure 3.**
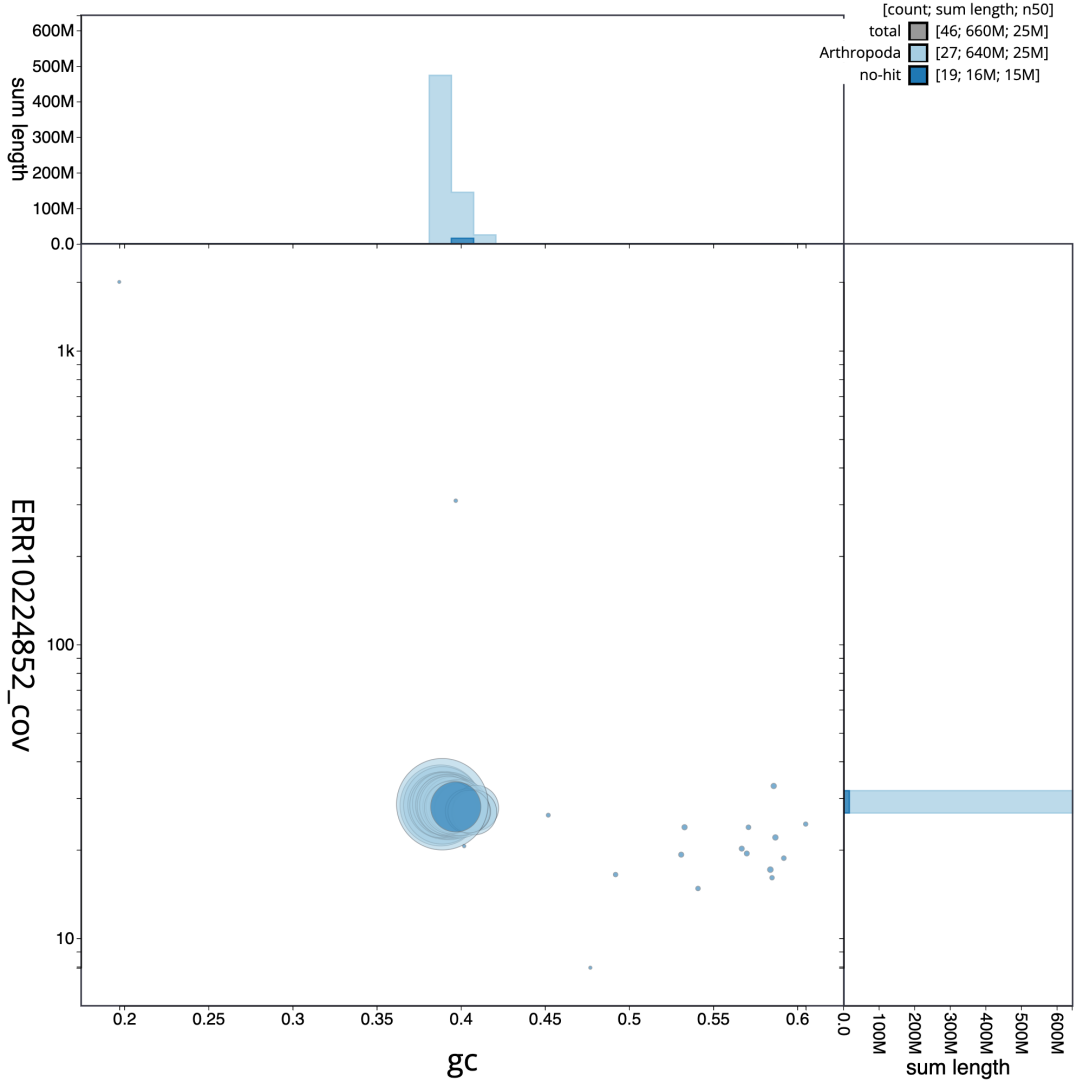
Genome assembly of *Epinotia bilunana*, ilEpiBilu1.1: BlobToolKit GC-coverage plot. Scaffolds are coloured by phylum. Circles are sized in proportion to scaffold length. Histograms show the distribution of scaffold length sum along each axis. An interactive version of this figure is available at https://blobtoolkit.genomehubs.org/view/ilEpiBilu1.1/dataset/CAMRIR01/blob.

**Figure 4.**
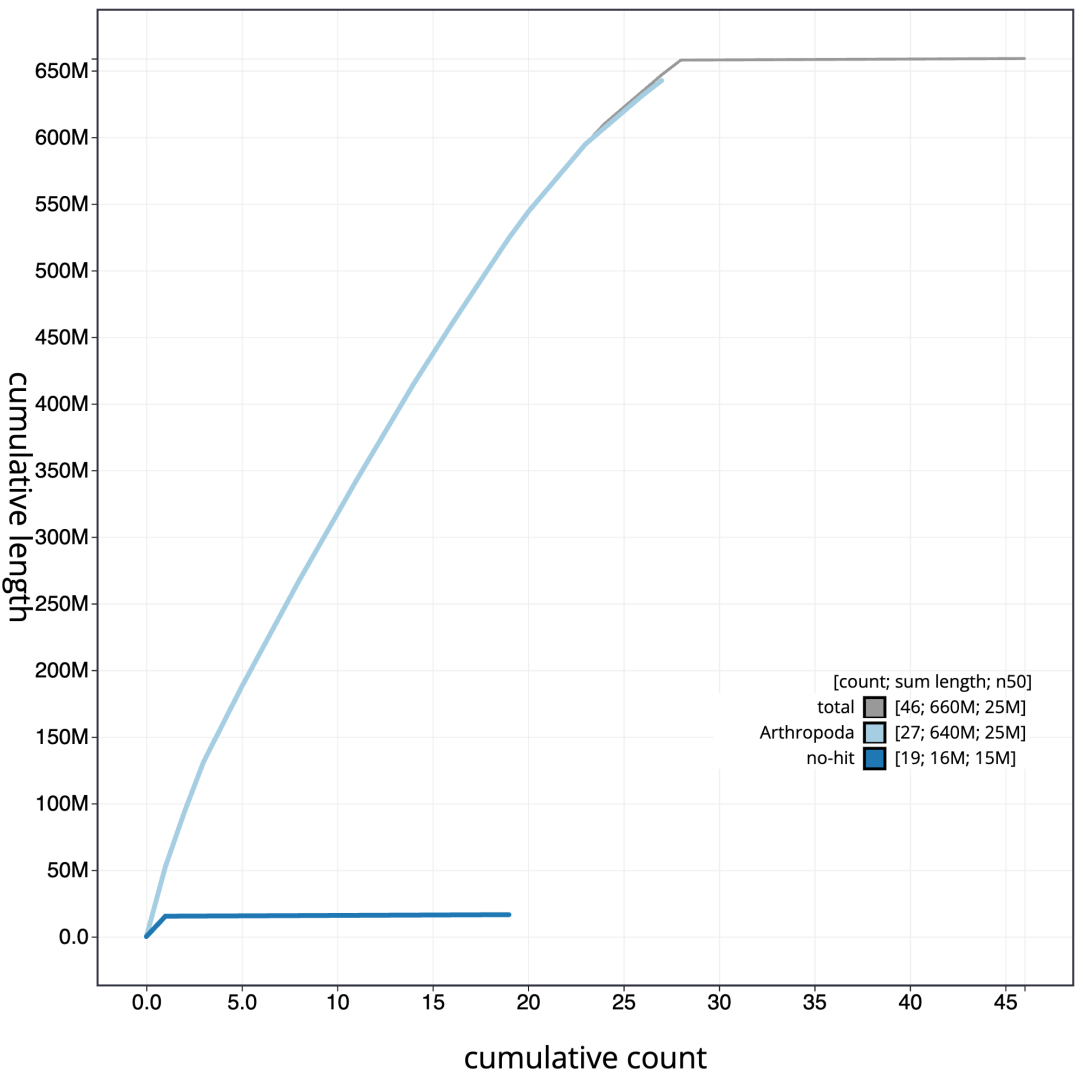
Genome assembly of *Epinotia bilunana*, ilEpiBilu1.1: BlobToolKit cumulative sequence plot. The grey line shows cumulative length for all scaffolds. Coloured lines show cumulative lengths of scaffolds assigned to each phylum using the buscogenes taxrule. An interactive version of this figure is available at https://blobtoolkit.genomehubs.org/view/ilEpiBilu1.1/dataset/CAMRIR01/cumulative.

**Figure 5.**
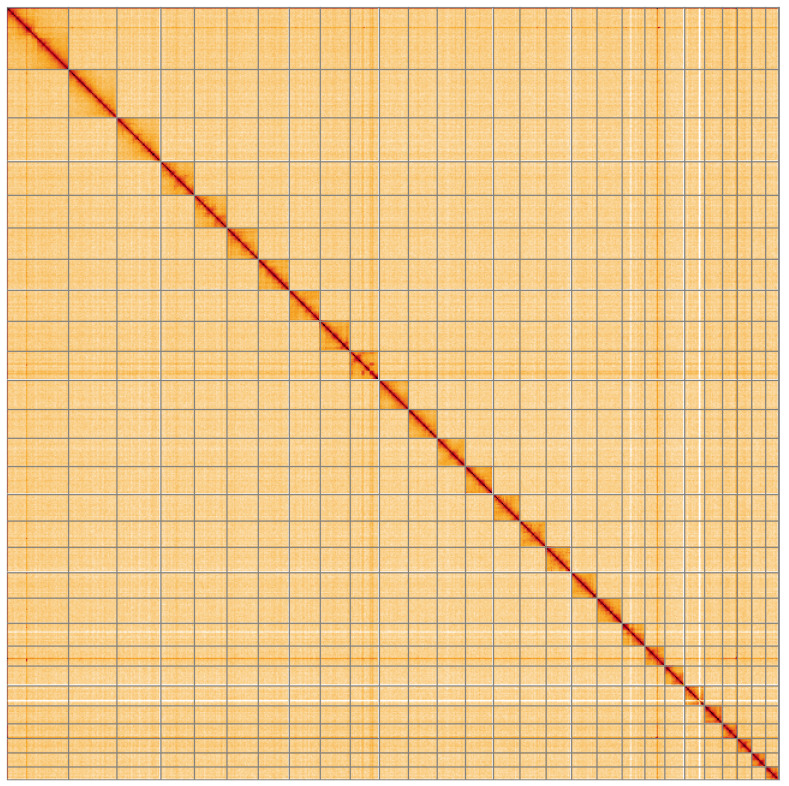
Genome assembly of *Epinotia bilunana*, ilEpiBilu1.1: Hi-C contact map of the ilEpiBilu1.1 assembly, visualised using HiGlass. Chromosomes are shown in order of size from left to right and top to bottom. An interactive version of this figure may be viewed at https://genome-note-higlass.tol.sanger.ac.uk/l/?d=VZ-7jSRjReSf9xze3k6gCw.

**Table 2.** Chromosomal pseudomolecules in the genome assembly of *Epinotia bilunana*, ilEpiBilu1.

INSDC accession	Chromosome	Size (Mb)	GC%
OX346253.1	1	41.16	38.8
OX346254.1	2	37.47	38.9
OX346255.1	3	28.58	39
OX346256.1	4	27.78	39.3
OX346257.1	5	26.72	38.8
OX346258.1	6	26.43	39.3
OX346259.1	7	26.39	38.9
OX346260.1	8	25.56	39.3
OX346261.1	9	24.85	39.5
OX346262.1	10	24.75	39.4
OX346263.1	11	24.51	39
OX346264.1	12	24.07	39.3
OX346265.1	13	23.86	39.2
OX346266.1	14	22.55	39.1
OX346267.1	15	22.38	39.2
OX346268.1	16	21.67	39.9
OX346269.1	17	21.65	39.5
OX346270.1	18	21.41	39.3
OX346271.1	19	19.43	39.8
OX346272.1	20	17	40
OX346273.1	21	16.96	39.5
OX346274.1	22	16.92	39.4
OX346275.1	23	15.34	39.7
OX346276.1	24	12.52	40.9
OX346277.1	25	12.28	40.8
OX346278.1	26	11.95	40.4
OX346279.1	27	11.03	40.5
OX346252.1	Z	52.66	38.9
OX346280.1	MT	0.02	19.9

The estimated Quality Value (QV) of the final assembly is 64.7 with *k*-mer based completeness of 100%, and the assembly has a BUSCO v5.3.2 ( Manni *et al.*, 2021) completeness of 97.9% (single = 97.4%, duplicated = 0.5%), using the lepidoptera_odb10 reference set ( *n* = 5,286).

Metadata for specimens, spectral estimates, sequencing runs, contaminants and pre-curation assembly statistics can be found at https://links.tol.sanger.ac.uk/species/1594293.

## Methods

### Sample acquisition and nucleic acid extraction

Two *Epinotia bilunana* specimens (ilEpiBilu1 and ilEpiBilu2) were collected from Wytham Woods, Oxfordshire (biological vice-county: Berkshire), UK (latitude 51.77, longitude –1.34) on 16 June 2021. The specimens were taken from woodland habitat by Douglas Boyes (University of Oxford) using a light trap. The specimens were identified by the collector and preserved on dry ice. Specimen ilEpiBilu1 (specimen number Ox001909) was used for genome sequencing, and ilEpiBilu2 (specimen number Ox001910) was used for Hi-C scaffolding.

DNA was extracted at the Tree of Life laboratory, Wellcome Sanger Institute (WSI). The ilEpiBilu1 sample was weighed and dissected on dry ice. Whole organism tissue was disrupted using a Nippi Powermasher fitted with a BioMasher pestle. High molecular weight (HMW) DNA was extracted using the Qiagen MagAttract HMW DNA extraction kit. HMW DNA was sheared into an average fragment size of 12–20 kb in a Megaruptor 3 system with speed setting 30. Sheared DNA was purified by solid-phase reversible immobilisation using AMPure PB beads with a 1.8X ratio of beads to sample to remove the shorter fragments and concentrate the DNA sample. The concentration of the sheared and purified DNA was assessed using a Nanodrop spectrophotometer and Qubit Fluorometer and Qubit dsDNA High Sensitivity Assay kit. Fragment size distribution was evaluated by running the sample on the FemtoPulse system.

### Sequencing

Pacific Biosciences HiFi circular consensus DNA sequencing libraries were constructed according to the manufacturers’ instructions. DNA sequencing was performed by the Scientific Operations core at the WSI on Pacific Biosciences SEQUEL II (HiFi) instrument. Hi-C data were also generated from tissue of ilEpiBilu2 using the Arima v2 kit and sequenced on the Illumina NovaSeq 6000 instrument.

### Genome assembly, curation and evaluation

Assembly was carried out with Hifiasm ( Cheng *et al.*, 2021) and haplotypic duplication was identified and removed with purge_dups ( Guan *et al.*, 2020). The assembly was then scaffolded with Hi-C data ( Rao *et al.*, 2014) using YaHS ( Zhou *et al.*, 2023). The assembly was checked for contamination as described previously ( Howe *et al.*, 2021). Manual curation was performed using HiGlass ( Kerpedjiev *et al.*, 2018) and Pretext ( Harry, 2022). The mitochondrial genome was assembled using MitoHiFi ( Uliano-Silva *et al.*, 2022), which runs MitoFinder ( Allio *et al.*, 2020) or MITOS ( Bernt *et al.*, 2013) and uses these annotations to select the final mitochondrial contig and to ensure the general quality of the sequence. To evaluate the assembly, MerquryFK was used to estimate consensus quality (QV) scores and *k*-mer completeness ( Rhie *et al.*, 2020). The genome was analysed within the BlobToolKit environment ( Challis *et al.*, 2020) and BUSCO scores ( Manni *et al.*, 2021; Simão *et al.*, 2015) were calculated. Table 3 contains a list of software tool versions and sources.

**Table 3.** Software tools and versions used.

Software tool	Version	Source
BlobToolKit	4.0.7	https://github.com/blobtoolkit/blobtoolkit
BUSCO	5.3.2	https://gitlab.com/ezlab/busco
Hifiasm	0.16.1-r375	https://github.com/chhylp123/hifiasm
HiGlass	1.11.6	https://github.com/higlass/higlass
Merqury	MerquryFK	https://github.com/thegenemyers/MERQURY.FK
MitoHiFi	2	https://github.com/marcelauliano/MitoHiFi
PretextView	0.2	https://github.com/wtsi-hpag/PretextView
purge_dups	1.2.3	https://github.com/dfguan/purge_dups
YaHS	yahs-1.1.91eebc2	https://github.com/c-zhou/yahs

### Ethics and compliance issues

The materials that have contributed to this genome note have been supplied by a Darwin Tree of Life Partner. The submission of materials by a Darwin Tree of Life Partner is subject to the Darwin Tree of Life Project Sampling Code of Practice. By agreeing with and signing up to the Sampling Code of Practice, the Darwin Tree of Life Partner agrees they will meet the legal and ethical requirements and standards set out within this document in respect of all samples acquired for, and supplied to, the Darwin Tree of Life Project. All efforts are undertaken to minimise the suffering of animals used for sequencing. Each transfer of samples is further undertaken according to a Research Collaboration Agreement or Material Transfer Agreement entered into by the Darwin Tree of Life Partner, Genome Research Limited (operating as the Wellcome Sanger Institute), and in some circumstances other Darwin Tree of Life collaborators.

## Data Availability

European Nucleotide Archive: *Epinotia bilunana* (crescent bell). Accession number PRJEB55885; https://identifiers.org/ena.embl/PRJEB55885. ( Wellcome Sanger Institute, 2022) The genome sequence is released openly for reuse. The *Epinotia bilunana* genome sequencing initiative is part of the Darwin Tree of Life (DToL) project. All raw sequence data and the assembly have been deposited in INSDC databases. The genome will be annotated using available RNA-Seq data and presented through the Ensembl pipeline at the European Bioinformatics Institute. Raw data and assembly accession identifiers are reported in Table 1.

## Author information

Members of the University of Oxford and Wytham Woods Genome Acquisition Lab are listed here: https://doi.org/10.5281/zenodo.4789928.

Members of the Darwin Tree of Life Barcoding collective are listed here: https://doi.org/10.5281/zenodo.4893703.

Members of the Wellcome Sanger Institute Tree of Life programme are listed here: https://doi.org/10.5281/zenodo.4783585.

Members of Wellcome Sanger Institute Scientific Operations: DNA Pipelines collective are listed here: https://doi.org/10.5281/zenodo.4790455.

Members of the Tree of Life Core Informatics collective are listed here: https://doi.org/10.5281/zenodo.5013541.

Members of the Darwin Tree of Life Consortium are listed here: https://doi.org/10.5281/zenodo.4783558.
