# Supplementary material for: The genome sequence of the Crescent Bell, Epinotia bilunana (Haworth, 1811)
Source: Wellcome Open Res. 2023 Apr 12;8:163. [Version 1] doi: 10.12688/wellcomeopenres.19334.1 (PMC11053349; doi:10.12688/wellcomeopenres.19334.1)
Supplement: Peer review history

# Reviewer report: Reviewer response for version 1

**DOI:** 10.21956/wellcomeopenres.21419.r79828

**Published:** 2024-04-26

**Authors:** Michael Hiller

**Affiliations:** 1 LOEWE-Centre for Translational Biodiversity Genomics (TBG), Senckenberg Nature Research Society, Frankfurt Am Main, Germany

This data note describes a long read based, high quality assembly of the Crescent Bell.The genome was sequenced using PacBio HiFi reads and scaffolded to chromosome-level using HiC data. 

 A primary and alternate assembly was produced. The primary assembly has a very high base accuracy and performs well in metrics that measure completeness. 

 Everything is done according to the high standards of DToL  and the methods are clearly described. 

 I only have one minor comment and a question.

 1) I couldn't find the contig N50 value mentioned in the text or in Table 1. This should be added.

 2) In the HiC plot, I wonder why the Z chromosome does not show a lighter background color compared to the autosomes. A male should only have one Z copy, while all autosomes are present in two copies. Is this related to the fact that the HiC data came from another individual (which was likely also a male)?

Are sufficient details of methods and materials provided to allow replication by others?

Yes

Is the rationale for creating the dataset(s) clearly described?

Yes

Are the datasets clearly presented in a useable and accessible format?

Yes

Are the protocols appropriate and is the work technically sound?

Yes

Reviewer Expertise:

genome assembly, comparative genomics

I confirm that I have read this submission and believe that I have an appropriate level of expertise to confirm that it is of an acceptable scientific standard.

# Reviewer report: Reviewer response for version 1

**DOI:** 10.21956/wellcomeopenres.21419.r56145

**Published:** 2023-04-28

**Authors:** Guillem Ylla

**Affiliations:** 1 Laboratory of Bioinformatics and Genome Biology, Uniwersytet Jagiellonski w Krakowie, Kraków, Lesser Poland Voivodeship, Poland

This article, authored by Douglas Boyes, and James Hammond, titled “The genome sequence of the Crescent Bell, *Epinotia biluana* (Haworth, 1811)”, as clearly stated in the title describes the release of the chromosome-level genome assembly of the moth *Epinotia biluana*. The article is concise, well written, and contains the information expected from a genome release publication.

 Materials and methods are exhaustively reported, with the software versions reported in Table 3.

 The raw data and the final assembly file have been deposited in public repositories and the accession identifiers are duly provided in the article.

 I only have a few minor comments:

- The rationale for sequencing this genome is not clearly stated. A few lines regarding the relevance of this species and the potential interest of this genome could be interesting.
- The contig N50 is described to be 247.5Mb in the text, but table 1 shows 24.7Mb. I am afraid there must be a typo in the text.
- It is not clear to me what point is Figure 3 intended to make, what information the reader should get from it, and how to interpret it. The figure is not specifically mentioned in the text (only referred to it within “Figure 1 – Figure 5”). Also, in the legend, it says that it has been colored by phylum, but it is not clear how the phylum assignment was done.

Are sufficient details of methods and materials provided to allow replication by others?

Yes

Is the rationale for creating the dataset(s) clearly described?

Partly

Are the datasets clearly presented in a useable and accessible format?

Yes

Are the protocols appropriate and is the work technically sound?

Yes

Reviewer Expertise:

My areas of expertise include bioinformatics, evolution, and insect genomics.

I confirm that I have read this submission and believe that I have an appropriate level of expertise to confirm that it is of an acceptable scientific standard.
